# Right ventricular strain assessment by cardiovascular magnetic resonance myocardial feature tracking allows optimized risk stratification in Takotsubo syndrome

**DOI:** 10.1371/journal.pone.0202146

**Published:** 2018-08-29

**Authors:** Thomas Stiermaier, Torben Lange, Amedeo Chiribiri, Christian Möller, Tobias Graf, Uwe Raaz, Adriana Villa, Johannes T. Kowallick, Joachim Lotz, Gerd Hasenfuß, Holger Thiele, Andreas Schuster, Ingo Eitel

**Affiliations:** 1 University Heart Center Lübeck, Medical Clinic II (Cardiology/Angiology/Intensive Care Medicine), University Hospital Schleswig-Holstein, Lübeck, Germany; 2 German Center for Cardiovascular Research (DZHK), partner site Hamburg/Kiel/Lübeck, Lübeck, Germany; 3 University Medical Center Göttingen, Department of Cardiology and Pneumology, Georg-August University, Göttingen, Germany; 4 German Center for Cardiovascular Research (DZHK), partner site Göttingen, Göttingen, Germany; 5 Division of Imaging Sciences and Biomedical Engineering, King’s College London, London, United Kingdom; 6 University Medical Center Göttingen, Institute for Diagnostic and Interventional Radiology, Georg-August University, Göttingen, Germany; 7 Heart Center Leipzig – University Hospital, Department of Internal Medicine/Cardiology, Leipzig, Germany; 8 University of Sydney, The Kolling Institute, Northern Clinical School, Royal North Shore Hospital, Department of Cardiology, Sydney, Australia; University of Palermo, ITALY

## Abstract

**Background:**

A substantial number of patients with Takotsubo syndrome (TTS) exhibit right ventricular (RV) dysfunction which has been associated with adverse outcome. The aim of this study was to assess the clinical and prognostic value of RV myocardial strain in TTS using cardiovascular magnetic resonance myocardial feature tracking (CMR-FT).

**Methods:**

CMR-FT was performed in a core laboratory to determine RV longitudinal strain in 134 TTS patients undergoing CMR in median 2 days after admission to 2 experienced centers. For comparison, RV involvement was evaluated by sole visual assessment concerning RV contraction abnormalities. Both approaches were analyzed regarding their long-term prognostic value.

**Results:**

The peak global RV longitudinal strain was in median -19%. Segmental analyses located contraction abnormalities primarily in the apical parts of the right ventricle. Sole visual assessment identified 38 patients (28%) with RV involvement. These patients showed a numerically higher long-term mortality without reaching statistical significance (17.1% versus 10.5%; hazard ratio 1.38 [95% confidence interval 0.49–3.88]; p = 0.31). The optimal RV strain cutoff value for risk prediction was -17.24%. Stratification according to this threshold categorized 41% of TTS patients (n = 55) in the high-risk group which demonstrated a significantly increased long-term mortality compared to patients with preserved global RV strain (20.0% versus 7.0%; hazard ratio 2.98 [95% confidence interval 1.02–8.73]; p = 0.03).

**Conclusions:**

The assessment of RV myocardial strain using CMR-FT enables an accurate evaluation of RV involvement in TTS and represents a promising approach for optimized risk stratification.

## Introduction

Takotsubo syndrome (TTS) is a form of acute heart failure due to circumscribed left ventricular (LV) contraction abnormalities constituting the morphological picture of apical, midventricular, or basal ballooning. [1–3] In addition, right ventricular (RV) involvement has been reported in a substantial number of TTS patients. [3–10] Although cardiac function recovers completely within several days to weeks, considerable mortality rates and a variety of potentially life-threatening complications have been reported in TTS populations. [11–14] In a large TTS registry, the death rate was estimated to be 5.6% per patient-year. [12] Consequently, recent research efforts focused mainly on prognostic factors in TTS and identified visual assessment of RV ballooning, among others, as a potential tool for optimized risk stratification. Involvement of the right ventricle was associated with prolonged hospital stay and increased short- and long-term adverse events in several investigations. [4–8] Therefore, evaluation of RV dysfunction may play a key role to guide the management of TTS patients. However, estimation of RV function using transthoracic echocardiography can be challenging since measuring RV area change in a single plane may be error-prone given the complex geometry of the right ventricle. Moreover, tricuspid annular plane systolic excursion (TAPSE) does not account for contraction abnormalities of the mid or apical RV segments and is influenced by basal hyperkinesia. Assessment of myocardial strain has shown to overcome some of these shortcomings in TTS patients [15] but still echocardiography is limited by the dependency on acoustic windows and operator experience. Cardiovascular magnetic resonance (CMR) imaging allows for a detailed and high-resolution illustration of regional LV and RV wall motion abnormalities in patients with TTS. Furthermore, CMR myocardial feature tracking (CMR-FT) has been introduced recently as a new method to quantify contractile dysfunction by tracking myocardial borders and following them over time similar to speckle tracking echocardiography. [16, 17] This technique offers great potential for a more accurate and less operator dependent evaluation of RV performance compared to sole visual evaluation and/or calculation of ejection fraction. Aim of the present study was therefore to determine CMR-FT derived RV myocardial strain in a large cohort of patients with TTS and to assess the clinical and prognostic value of this novel approach.

## Materials and methods

### Study population

This international multi-center study included 141 patients with TTS presenting to the University of Leipzig—Heart Center (n = 125) or the King’s College London—St. Thomas’ Hospital (n = 16). All patients met the established diagnostic criteria for TTS: (a) hypokinesia, akinesia, or dyskinesia of the apical and/or midventricular or basal LV segments extending beyond a single epicardial vessel distribution territory with complete recovery during follow-up; (b) absence of acute plaque rupture or significant obstructive coronary artery disease explaining the extent of wall motion abnormalities; (c) new electrocardiographic changes or modest elevation in cardiac troponin levels; and (d) absence of pheochromocytoma and myocarditis. [2] Moreover, all patients fulfilled the previously published CMR criteria to confirm TTS and exclude important differential diagnoses with similar clinical appearance (e.g. myocardial infarction with spontaneous lysis of thrombus or myocarditis). [3] Clinical follow-up included an outpatient visit within 6 months to document complete recovery of LV dysfunction. Follow-up CMR data after normalization of systolic function were available in 21 TTS patients undergoing serial CMR scans. Long-term clinical outcome was assessed during regular outpatient visits or with structured telephone interviews with the patients, relatives, and treating physicians performed by personnel unaware of the CMR scan results. All events were verified via medical records and adjudicated by a clinical events committee.

The study was conducted according to the principles of the Declaration of Helsinki and approved by the institutional review boards of the participating study centers (University of Leipzig, King’s College London). Written informed consent was obtained.

### Cardiovascular magnetic resonance image acquisition and analysis

**Fig 1:**
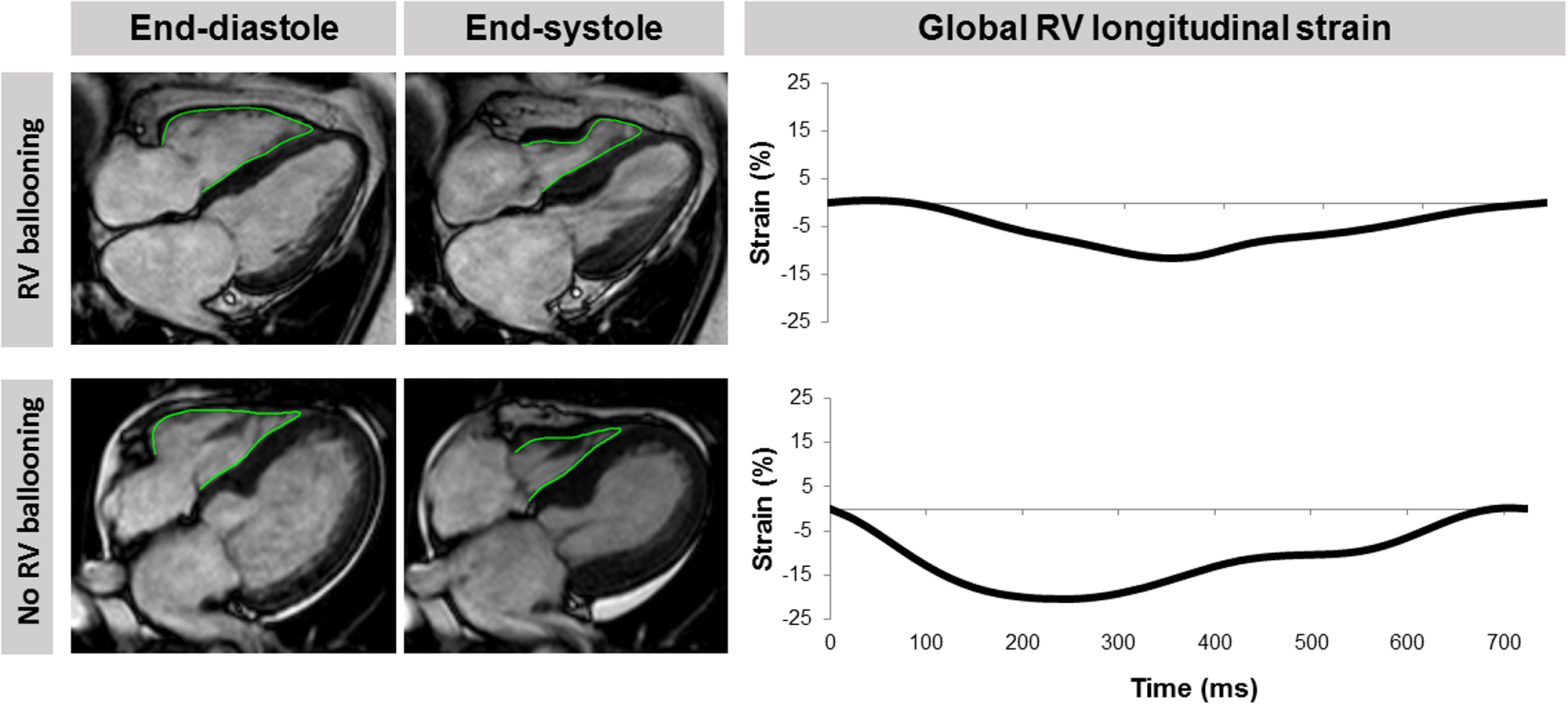
Analysis of right ventricular longitudinal strain. CMR-FT of long-axis 4-chamber views to quantify longitudinal RV strain in a TTS patient with and without RV involvement. RV = right ventricular.

CMR imaging was performed on 1.5- or 3.0-T magnetic resonance scanners using a standardized protocol which included (a) ECG-gated balanced steady state—free precession (SSFP) sequences for functional analyses; (b) T2-weighted triple short-tau inversion recovery images for edema assessment; and (c) early as well as late gadolinium enhancement sequences for the detection of inflammation or fibrosis/necrosis. [3, 18, 19] One experienced investigator analyzed all scans offline with certified CMR evaluation software (cmr42, Circle Cardiovascular Imaging Inc, Calgary, Alberta, Canada) and checked the CMR criteria for TTS. [3] RV involvement and the presence of tricuspid valve regurgitation were determined in long axis 4-chamber ECG-gated balanced SSFP sequences. Only scans with sufficient image quality to derive valid CMR-FT data were included in the study. Sole visual assessment was performed in a core laboratory at the University Heart Center Lübeck. Patients were categorized into RV involvement or no RV involvement according to the presence of regional RV contraction abnormalities causing systolic dysfunction. Mostly, RV wall motion abnormalities affect the apical RV segments in TTS patients while mid and basal contraction is preserved or hyperkinetic. This phenomenon has been described as “reverse McConnell’s sign” and was the basis for the definition of RV involvement in previous TTS trials. [4, 7, 20] All images were evaluated independently by 2 investigators blinded to clinical patient data. Any disagreements were resolved by consensus. CMR-FT was performed in a core laboratory with vast experience in this field at the University Medical Center Göttingen using dedicated evaluation software that has been extensively validated and used in numerous studies (2D CPA MR, Cardiac Performance Analysis, Version 1.1.2, TomTec Imaging Systems, Unterschleissheim, Germany). [16, 17, 21–24] RV endocardial borders were manually traced at end-diastole using a point-and-click approach (Fig 1). Subsequently, the software’s automatic border tracking algorithm was applied which tracks image features throughout the whole cardiac cycle. Accurate tracking was assured by visual review of all borders and manual adjustments with consequent reapplication of the algorithm if necessary. Peak RV longitudinal strain and time-to-peak (TTP) strain durations were determined. Final values were based on the average of 3 repeated, independent analyses. [24] The results are reported globally for the whole right ventricle as well as on a segmental basis at apical, midventricular, and basal levels.

### Statistical analysis

Categorical variables are presented as number (percentage) of patients and were compared with the Chi-square test. The Shapiro-Wilk test was used to test continuous variables for normal distribution. If normally distributed, variables are reported as mean ± standard deviation and were compared with the student *t*-test. Non-normally distributed continuous data are presented as median (interquartile range; IQR). Testing for differences was performed with the Mann-Whitney U test. Since CMR-FT data were non-normally distributed, the Kruskal-Wallis test was used to compare TTS patients with different LV ballooning patterns. Correlation between LV ejection fraction and global RV strain was analyzed with the Spearman method. Comparison of global RV strain between the acute phase and after recovery within the cohort of patients with serial CMR data was performed with the Wilcoxon signed rank test. The prognostic implications of RV involvement were assessed by dichotomizing the study population according to (a) visual assessment and (b) global RV strain. The optimal cutoff point for global RV strain was defined with the Youden index after application of receiver operating characteristic curve. Long-term mortality was compared between groups using Chi-square test as well as Kaplan-Meier analysis and log-rank testing. Furthermore, hazard ratios (HR) and corresponding 95% confidence intervals (CI) were calculated. Statistical analyses were performed with SPSS (version 17.0; SPSS Inc., Chicago, IL, USA). A two-sided probability ≤0.05 was considered statistically significant.

## Results

Of the 141 eligible TTS cases, 7 patients (5%) were excluded due to severe breathing artefacts in long-axis 4-chamber ECG-gated balanced SSFP images that did not allow for reliable tracking. Consequently, the final study population consisted of 134 TTS patients. Coronary angiography was performed in all patients and excluded significant obstructive coronary artery disease. CMR was performed in median 2 days (IQR 2, 4) after initial presentation and confirmed TTS in all patients.

### Main patient characteristics

Baseline characteristics are summarized in Table 1. The study cohort was predominantly female with a median age of 71 years, therefore reflecting a typical TTS population. The majority of patients showed typical apical LV ballooning (69.4%) and 64.2% had a preceding stressful trigger. Systolic LV function recovered completely in all patients during follow-up.

**Table 1:** Main patient characteristics.

Variable	All patients (n = 134)	RV strain > -17.24% (n = 55)	RV strain ≤ -17.24% (n = 79)	p
Age (years)	71 (61, 77)	72 (63, 79)	70 (61, 77)	0.17
Female sex	112 (83.6)	49 (89.1)	63 (79.7)	0.15
**Cardiovascular risk factors**				
Current smoking	21 (15.7)	10 (18.2)	11 (13.9)	0.55
Hypertension	104 (77.6)	44 (80.0)	60 (75.9)	0.77
Hypercholesterolemia	32 (23.9)	14 (25.5)	18 (22.8)	0.78
Diabetes mellitus	30 (23.4)	13 (23.6)	17 (21.5)	0.83
Killip-class 3/4 on admission	7 (5.2)	4 (7.3)	3 (3.8)	0.37
Cardiogenic shock	5 (3.7)	3 (5.5)	2 (2.5)	0.38
Days of hospitalization	4 (3, 6)	5 (3, 7)	4 (3, 6)	0.77
**Stressful event**	86 (64.2)	43 (78.2)	43 (54.4)	**<0.01**
Emotional	37 (27.6)	15 (27.3)	22 (27.8)	0.94
Physical	49 (36.6)	28 (50.9)	21 (26.6)	**<0.01**
**Ballooning pattern**				
Apical	93 (69.4)	93 (69.4)	51 (64.6)	0.15
Midventricular	39 (29.1)	12 (21.8)	27 (34.2)	0.12
Basal	2 (1.5)	1 (1.8)	1 (1.3)	0.80
Initial LV-EF (%)	46.1 ± 8.7	44.1 ± 8.4	47.7 ± 8.7	**0.03**
Follow-up LV-EF (%)	60.4 ± 7.1	60.2 ± 7.4	60.5 ± 7.0	0.79

Data are presented as n (%), median (IQR), or mean ± standard deviation. P-values were calculated for the comparison between patients with a global RV strain > -17.24% versus ≤ -17.24%. Numbers in bold type indicate a significant difference. EF = ejection fraction; LV = left ventricular; RV = right ventricular.

### Right ventricular myocardial strain in Takotsubo syndrome

**Fig 2:**
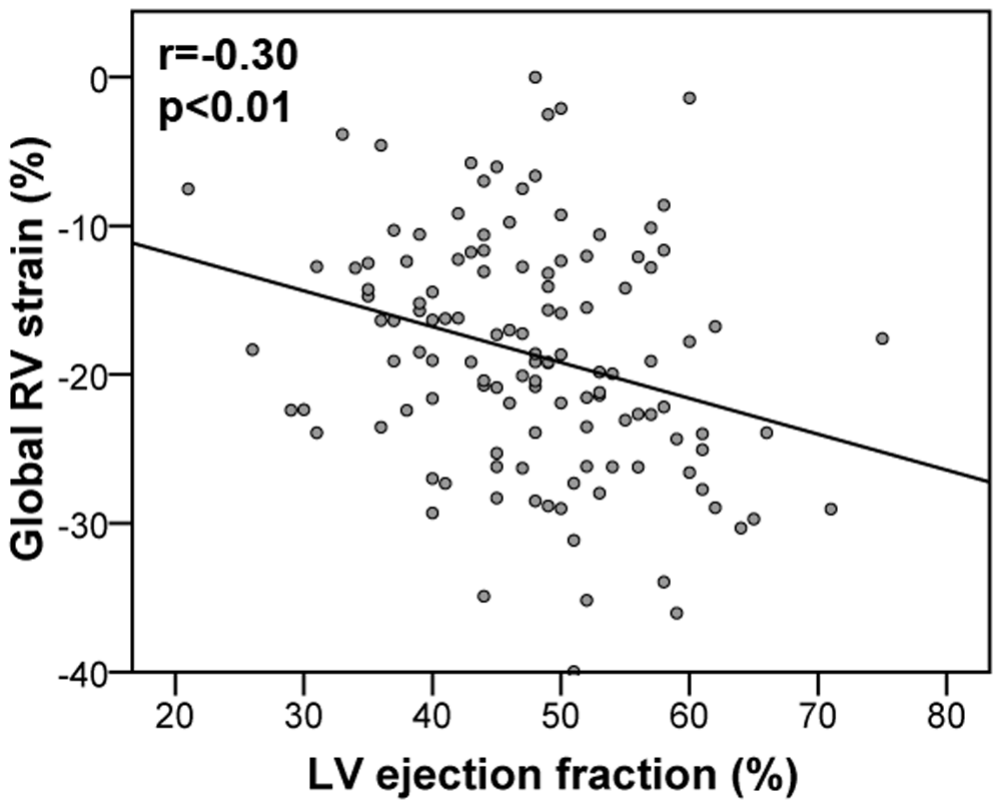
Global right ventricular strain plotted against left ventricular ejection fraction. Global RV strain correlated significantly with LV ejection fraction determined in CMR imaging. LV = left ventricular; RV = right ventricular.

Global and segmental RV longitudinal strain values are reported in Table 2. The peak global RV strain was in median -19% (IQR -13, -25) with a TTP of 368ms (331, 408). Segmental analyses revealed a more severe impairment of apical compared to mid or basal parts of the right ventricle, which was particularly obvious in the lateral segments corresponding to the free RV wall. Variations between TTS patients with different LV ballooning patterns were only marginal (Table 2). A moderate correlation reaching statistical significance was observed between global RV strain and LV ejection fraction determined in CMR imaging (Fig 2).

**Table 2:** Right ventricular myocardial strain in Takotsubo syndrome.

	Total cohort (n = 134)	Left ventricular ballooning pattern			p
		Apical (n = 93)	Midventricular (n = 39)	Basal (n = 2)	
**Global**					
Peak strain (%)	-19 (-13, -25)	-18 (-12, -25)	-21 (-16, -25)	-18 (-13, -23)	0.44
TTP strain (ms)	368 (331, 408)	318 (268, 385)	313 (264, 359)	456 (410, 502)	**<0.01**
**Basal septal**					
Peak strain (%)	-16 (-9, -24)	-16 (-10, -24)	-16 (-9, -26)	-7 (-13, -1)	0.33
TTP strain (ms)	365 (288, 459)	364 (277, 463)	382 (288, 459)	445 (312, 579)	0.80
**Mid septal**					
Peak strain (%)	-24 (-15, -34)	-23 (-13, -35)	-25 (-19, -32)	-26 (-16, -35)	0.50
TTP strain (ms)	329 (280, 425)	326 (272, 435)	335 (288, 413)	391 (313, 469)	0.75
**Apical septal**					
Peak strain (%)	-11 (-6, -18)	-11 (-6, -20)	-12 (-7, -17)	-24 (-14, -35)	0.42
TTP strain (ms)	386 (278, 495)	377 (264, 488)	398 (296, 513)	515 (494, 536)	0.25
**Apical lateral**					
Peak strain (%)	-17 (-10, -26)	-17 (-10, -26)	-20 (-10, -26)	-19 (-32, -6)	0.85
TTP strain (ms)	383 (297, 465)	398 (288, 478)	358 (299, 445)	506 (301, 712)	0.55
**Mid lateral**					
Peak strain (%)	-26 (-13, -35)	-22 (-11, -35)	-28 (-21, 35)	-50 (-45, -55)	**0.02**
TTP strain (ms)	313 (253, 385)	318 (251, 404)	308 (254, 372)	245 (189, 301)	0.48
**Basal lateral**					
Peak strain (%)	-23 (-15, -33)	-24 (-16, -34)	-20 (-14, -28)	-18 (-1, -35)	0.58
TTP strain (ms)	332 (268, 423)	330 (263, 395)	348 (286, 466)	637 (313, 961)	0.26

Data are presented as median (IQR). P-values were calculated for the comparison between different ballooning patterns. Numbers in bold type indicate a significant difference. TTP = time-to-peak

### Recovery of right ventricular myocardial strain

**Fig 3:**
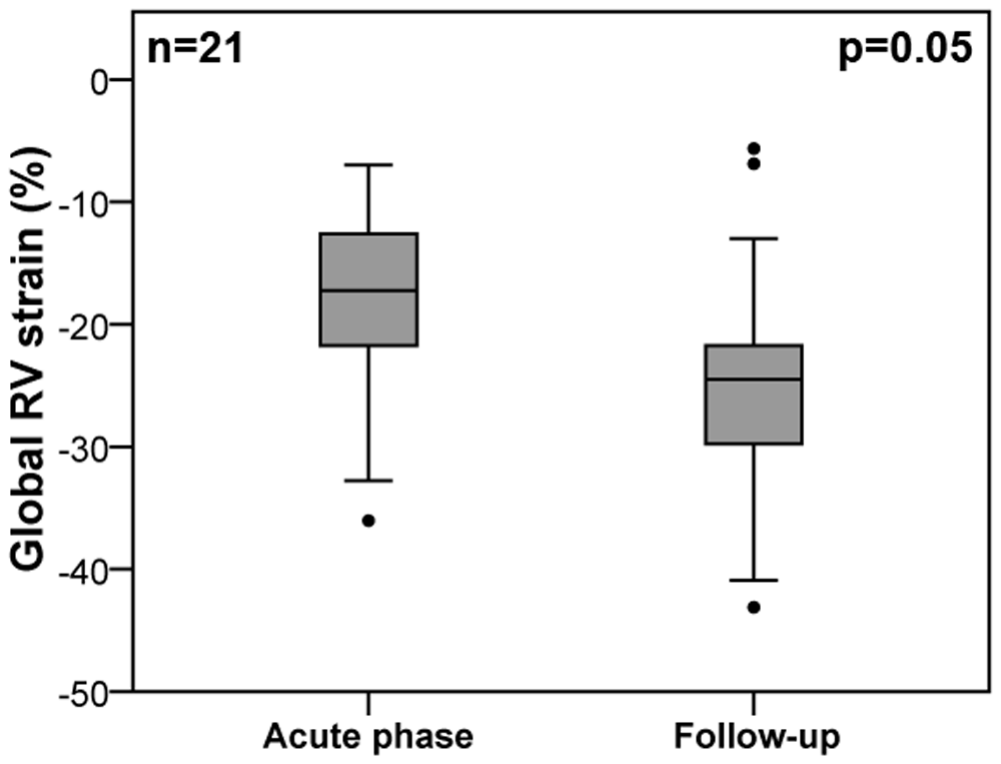
Recovery of global right ventricular strain in Takotsubo syndrome. Box (25^th^ percentile, median, 75^th^ percentile) and whisker (10^th^ and 90^th^ percentile) plots illustrating recovery of global RV strain during follow-up. RV = right ventricular.

Serial CMR data in median 3.5 months (IQR 3, 5) after the initial event were available in 21 TTS patients. Global RV longitudinal strain improved from -17% (IQR -13, -23) to -25% (IQR -21, -30; p = 0.05; Fig 3). TTP global RV strain remained largely unchanged at follow-up (315 ms [IQR 264, 410] versus 344 ms [IQR 320, 396]; p = 0.31).

### Right ventricular involvement according to visual assessment and myocardial strain

**Fig 4:**
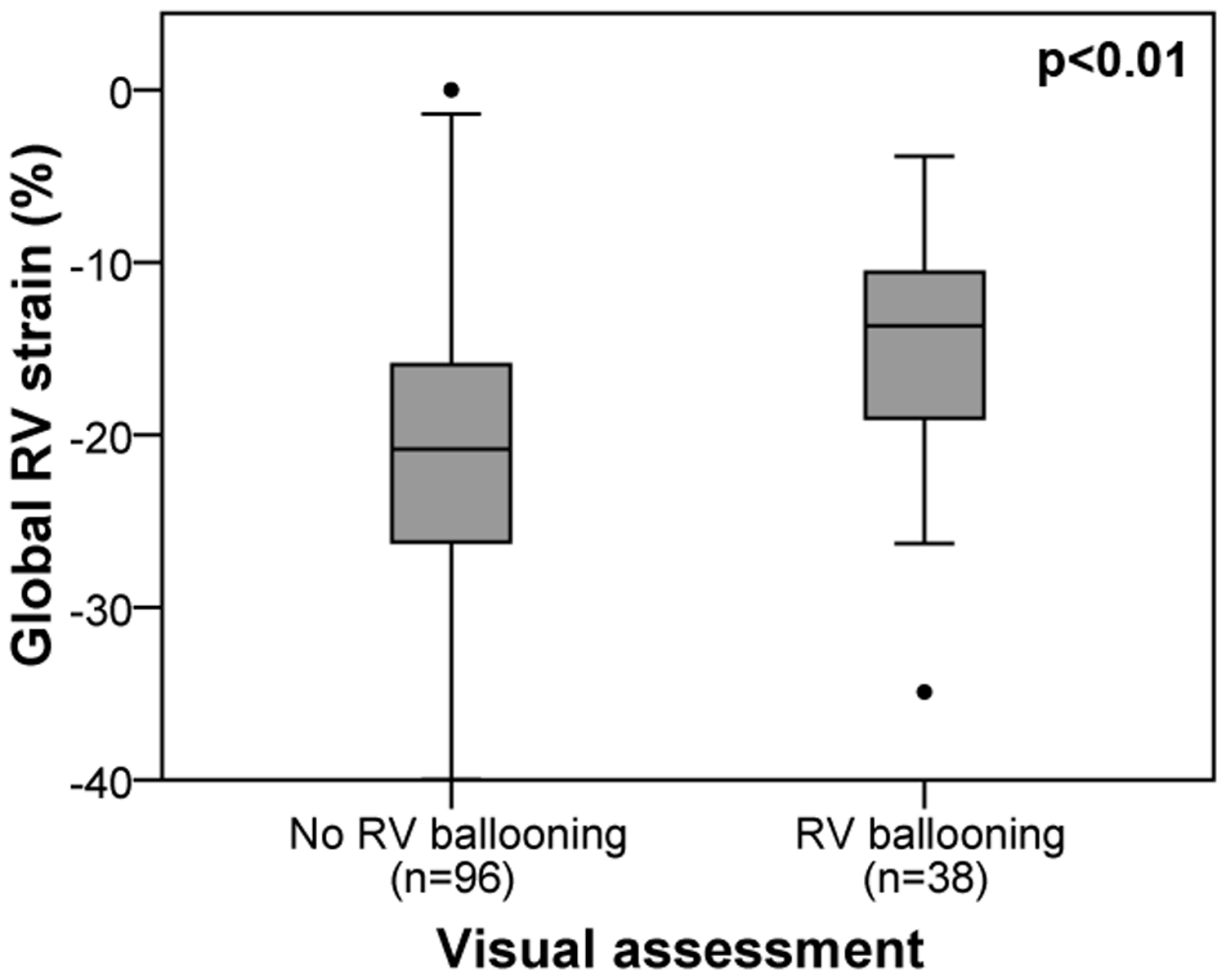
Global right ventricular strain in patients with and without right ventricular involvement according to visual assessment. Box (25^th^ percentile, median, 75^th^ percentile) and whisker (10^th^ and 90^th^ percentile) plots in TTS patients with and without RV ballooning according to sole visual assessment. RV = right ventricular.

Sole visual assessment identified RV involvement in 38 patients (28%). Consistently, these patients demonstrated significantly lower global RV strain values compared to patients without RV ballooning (-14% [IQR -10, -19] versus -21% [IQR -16, -26]; p<0.01; Fig 4). Using the Youden index, a global RV strain of -17.24% was identified as the best cutoff point for long-term mortality prediction. Stratification of the TTS population according to this threshold categorized 55 patients (41%) in the group with RV involvement. Patients with a global RV strain > -17.24% showed a higher prevalence of physical stressors (p<0.01) and a significantly lower initial LV ejection fraction (p = 0.03) compared to TTS patients with strain values below this threshold (Table 1). The incidence of tricuspid valve regurgitation was numerically higher in patients with biventricular ballooning without reaching statistical significance (38.2% versus 25.3%; p = 0.11). Transient tricuspid valve regurgitation with complete recovery during follow-up was observed in 1 patient with right ventricular involvement during acute TTS. The other patients showed stable findings.

### Prognostic value of right ventricular myocardial strain in Takotsubo syndrome

**Fig 5:**
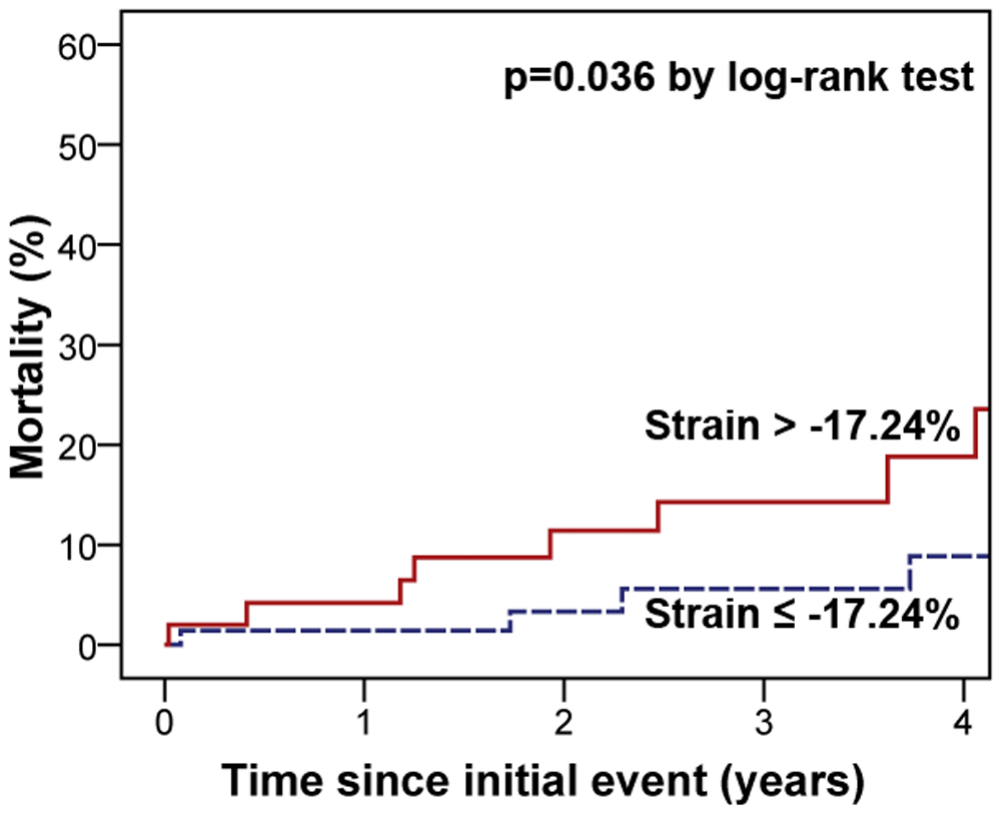
Kaplan-Meier analysis for long-term mortality according to global right ventricular strain. Patients were stratified according to global RV strain (optimal cutoff determined by the Youden index). TTS patients with a strain value > -17.24% demonstrated a significantly higher mortality compared to patients with a strain of ≤ -17.24%.

Long-term follow-up data were available in 121 of the 134 patients (90%) after a median of 3.1 years (IQR 1.7, 5.6). A total of 15 patients died (11.2%) due to non-cardiovascular (n = 6), cardiovascular (n = 4), or unknown causes of death (n = 5). Compared to patients without RV ballooning in visual assessment, long-term mortality was numerically higher in patients with RV involvement without reaching statistical significance (6/35 [17.1%] versus 9/86 [10.5%]; HR 1.38 [95% CI 0.49–3.88]; p = 0.31). Using the cutoff for global RV strain resulted in a significantly increased long-term mortality in TTS patients with a strain value > -17.24% (10/50 [20.0%]) compared to ≤ -17.24% (5/71 [7.0%]; HR 2.98 [95% CI 1.02–8.73]; p = 0.03; Fig 5). This result was mainly driven by an increased number of cardiovascular and unknown causes of death among patients with a RV strain > -17.24% (7/50 [14.0%] versus 2/71 [2.8%]; p = 0.02) while non-cardiovascular mortality did not differ significantly (3/50 [6.0%] versus 3/71 [4.2%]; p = 0.66).

## Discussion

The present study is the first to investigate RV myocardial strain in patients with TTS using CMR-FT. The results suggest that this novel technique allows for a reliable and optimized detection of RV involvement in patients with TTS and a more accurate risk stratification compared to sole visual assessment.

### Right ventricular Takotsubo syndrome

Since its first description more than 25 years ago, TTS has been increasingly recognized worldwide and research efforts have been directed to enhance the understanding of the disease. [1] In the course of time, atypical variants with midventricular or basal LV ballooning and RV forms have been described. [1–3, 25, 26] Mostly, RV ballooning is associated with LV TTS albeit cases with isolated RV TTS have been reported as well. [27] The underlying mechanisms of TTS and the reasons for RV involvement in some patients are not understood yet. The estimated prevalence of RV involvement in currently available literature varies considerably from <15% to as much as 50% of TTS patients. [3–10] The divergent frequencies could be attributed to different definitions of RV involvement, different sensitivities of the diagnostic modalities that have been used (echocardiography, CMR imaging), and different study populations with some investigators analyzing only typical apical TTS with a higher prevalence of RV involvement compared to non-apical forms. The observed frequency of 28% according to visual assessment in our study population is consistent with previous CMR studies. [3] Although this approach obviously identified TTS patients with true RV dysfunction which is illustrated by the significantly lower global RV strain in patients categorized as having RV involvement (Fig 4), the objective and accurate quantification of RV performance with strain analyses holds the potential to provide additional insights.

The feasibility of echocardiographic strain imaging to assess RV involvement in TTS has been investigated previously. [15] Our first study on CMR-FT confirms the applicability of this new technique in TTS patients and shows that contraction abnormalities are mainly located in the apical segments of the right ventricle and seem to be reversible during follow-up, similarly to LV dysfunction. However, the presence of RV involvement hardly changes the medical therapy of the individual patient because of the lack of specific treatment options. Therefore, from a clinician’s point of view the main benefit of a comprehensive RV evaluation is the potentially optimized risk stratification of TTS patients with implications for the duration of monitoring at intensive/intermediate care units, decision making for mechanical support devices, and the frequency of follow-up visits.

### Prognostic value of right ventricular involvement

The observation of severe complications and substantial mortality rates in various TTS populations challenged the previously prevalent opinion of a benign disease. [11–13] Efforts to develop risk prediction models identified RV involvement as a determinant of increased morbidity and mortality in TTS. [4–8] In contrast, some smaller studies could not confirm the predictive value of RV ballooning for future cardiovascular events. [9, 10] Likewise, in our study mortality rates did not differ significantly between patients with and without RV involvement in visual assessment. However, stratification according to the RV strain threshold of -17.24% categorized considerably more TTS patients in the high-risk group (41%) compared to visual evaluation (28%) and resulted in an almost threefold increased risk of death in patients with impaired RV strain. These findings suggest that CMR-FT derived RV myocardial strain allows for a more accurate risk assessment in TTS patients. Furthermore, CMR-FT may be useful to identify patients at risk for RV and/or LV thrombus formation which has been reported in TTS. [28, 29] Affirming and expanding the prognostic implications of RV dysfunction in TTS raises the question regarding the causal connections with adverse events. Several studies reported increased comorbidity in TTS patients with RV involvement suggesting a priori a limited prognosis. [4, 7] This aspect may impact the acute phase of the disease and could explain non-cardiovascular mortality during long-term follow-up. Furthermore, RV involvement has been associated with a more severe impairment of LV function in previous investigations. [7, 15] Our data confirm these observations by showing a correlation between LV ejection fraction and RV myocardial strain. Therefore, patients with biventricular ballooning may be particularly prone to a severe clinical course with heart failure and/or cardiogenic shock given the higher degree of LV dysfunction which is further compromised by the reduced LV preload due to RV dysfunction. [9] Also noteworthy, physical stressors—a known predictive factor in TTS—were more prevalent in patients with RV ballooning in our study. [30] Nevertheless, a definite statement concerning the causes of increased mortality in TTS patients with RV involvement requires clarifying the still unexplained etiology of the disease. Neither of the previously proposed mechanisms fully captures all aspects of TTS and particularly the reasons for the local distribution of the contraction abnormalities with or without RV involvement are not understood yet. Previous studies reported tricuspid valve regurgitation as a consequence of right ventricular involvement in patients with TTS. [31, 32] However, our systematic analysis did not show a significantly elevated rate of tricuspid valve regurgitation in patients with biventricular ballooning and transient valve insufficiency potentially related to right ventricular dysfunction was only observed in one patient.

### Limitations

The present study is subject to an unavoidable selection bias regarding stable patients without contraindications to undergo CMR imaging. This resulted in a low-risk study population with a moderate number of events during follow-up which does not entirely reflect the actual prognosis of TTS patients. Nevertheless, since fewer events generally impede risk prediction our results underscore the potential of the utilized approach. The observed RV strain cutoff value for increased risk has to be validated in independent TTS cohorts. The lack of a healthy control population impedes the comparative interpretation of the observed RV strain values and recovery of myocardial deformation was only analyzed in a small sub-population of patients with follow-up CMR data. These aspects require further evaluation in future trials. The presence of tricuspid valve regurgitation was assessed in 4-chamber view CMR images which might be inferior compared to systematic echocardiographic evaluation in multiple views. Several software solutions to perform CMR-FT are available and demonstrated some differences in strain measurements. [16, 24] However, an experienced core laboratory performed all analyses in this study utilizing software which is extensively validated and has been used in the majority of previous CMR-FT trials.

## Conclusions

The assessment of RV myocardial deformation using CMR-FT enables an accurate evaluation regarding RV involvement in patients with TTS. Furthermore, this novel technique represents a promising approach for optimized risk stratification in TTS based on RV myocardial strain.
